# A recurrent mutation of GJB6 in a big Chinese family with Hidrotic ectodermal dysplasia

**DOI:** 10.1186/s41065-020-00148-8

**Published:** 2020-08-25

**Authors:** Yi Zhan, Shuaihantian Luo, Zixin Pi, Guiying Zhang

**Affiliations:** Department of Dermatology, The Second Xiangya Hospital of Central South University, Changsha, 410011 China

**Keywords:** Hidrotic ectodermal dysplasia, Gene mutations, Sequence analysis, GJB6

## Abstract

Hidrotic ectodermal dysplasia (HED) is a rare inherited syndrome characterised by nail dystrophy, palmoplantar hyperkeratosis and alopecia. Four mutations (p.G11R, p.A88V, p.V37E and p.D50N) in gap junction beta 6 (GJB6) gene, which codes connexin30 protein, have been found to cause HED in different populations. Here, we reported a big Chinese family in which 24 patients over five generations were suffered with HED. Sequence analysis identified all 24 patients carry a recurrent missense mutation c.263C > T (p.A88V) in GJB6. Our results reveal gene testing of GJB6 is important for diagnosis, prenatal diagnosis and future gene treatment of HED.

## Introduction

Hidrotic ectodermal dysplasia (HED) (OMIM: 129500), also called Clouston syndrome, is a rare autosomal dominant inherited syndrome [1]. In year of 1895, Nicolle and Hallipre first reported this disease [2]. HED occurs worldwide with a very low frequency of 1:100000 [3], while it is high frequent in French-Canadians, which may result from founder effect [4]. The main clinical manifestations of HED are nail dystrophy, alopecia and palmoplantar hyperkeratosis with normal sweat glands and teeth. Besides, eye abnormalities, sensorineural hearing loss, oral leukoplakia may also happen in some sporadic cases [1, 2, 5]. Previous studies have identified that mutations in the gap junction beta 6 (*GJB6*) gene encoding connexin30 (Cx30) protein was the main cause of HED. Based on gene analysis in different populations, it has been demonstrated that at least four mutation loci (p.G11R, p.A88V, p.V37E and p.D50N) in *GJB6* can cause this disorder jointly or independently [6–9]. In this article, we reported a big Chinese family in which 24 patients over five generations were suffered with HED. The proband is a 26-year-old young female. Sequence analysis revealed she and other 23 family members all carry a recurrent missense mutation p.A88V (c.263C > T) in *GJB6*. We presented this case to highlight the pathogenic role of p.A88V mutation in *GJB6* and emphasize the importance of gene testing in diagnosis, prenatal diagnosis and future gene treatment of HED.

## Case history

**Fig. 1:**
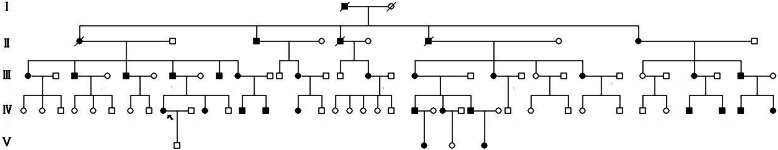
Pedigree of this family studied. The arrow indicates the proband

**Fig. 2:**
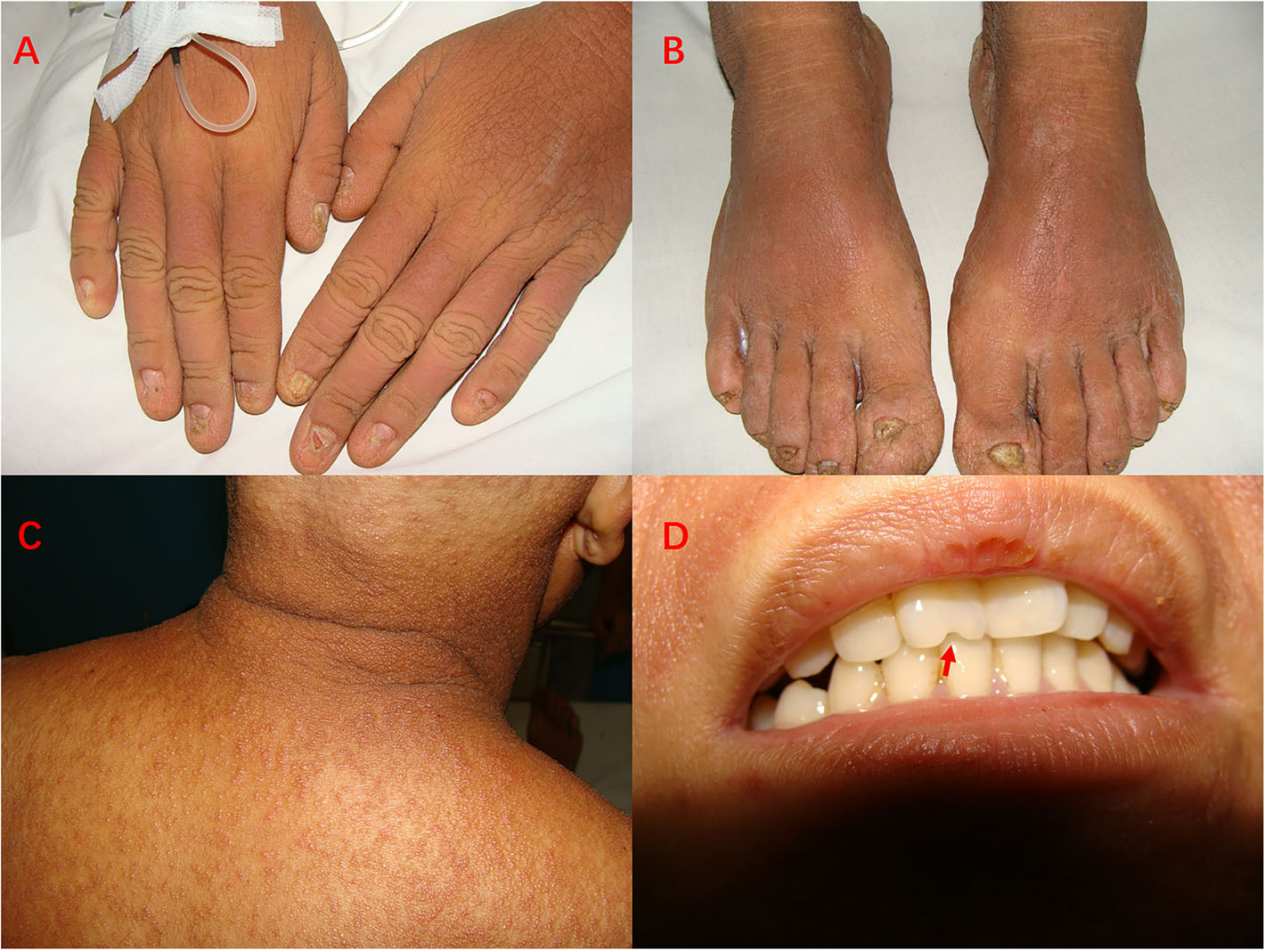
Clinical features of the proband. **a** The fingers’ nails of the proband are atrophic, thin and brittle. **b** The toes’ nails are short and thickened. **c** The skin has dense follicular hyperkeratotic papules. **d** One of the incisors is absence partly (red arrow)

The big Chinese HED family is from Hunan Province including 33 patients (16 males and 17 females) over five generations (Fig. 1). The autosomal dominate inheritance can be observed in pedigree. The proband (IV9) is a 26-year-old young woman. Physical examination revealed that her fingernails and toenails are atrophic, short, thickened and brittle with pterygium formation (Fig. 2a, b). The skin all over her body is thickened and hyperkeratosis with dense follicular hyperkeratotic papules (Fig. 2c). Her scalp and body’s hair were totally absent since she was born. Only one of the incisors is absence partly and other teeth are all normal (Fig. 2d). Sweating and cutaneous sensation are also normal. Skin biopsy of trunk showed hyperkeratosis with normal distribution of eccrine and sebaceous gland, but absence of hair follicles. Almost all the affected individuals have similar clinic features include nail dystrophy, palmoplantar hyperkeratosis and alopecia. Only two patients have hearing disorders (the proband and her father).

**Fig. 3:**
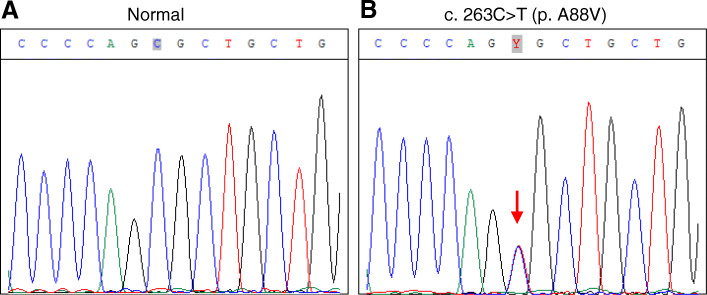
Molecular genetic analysis of the *GJB6* gene with a heterozygous mutation. **a** Normal *GJB6* sequence showing nucleotides 257–270. **b** The equivalent region from the affected individual with a heterozygous mutation c. 263C > T (arrow)

Next, we collected blood samples from 24 patients and 6 normal persons for gene testing. Sanger sequencing of DNA from the affected individuals were performed and a heterozygous single nucleotide transition, c.263C > T, which results in the amino acid substitution, p.A88V, within *GJB6* was identified in all available 24 patients (Fig. 3). None mutations were found in the *GJB6* gene in the other 6 healthy individuals.

## Discussion

Connexins are structurally transmembrane proteins that assemble to form gap junctions in human and vertebrate [1]. Gap junctions are essential for many physiological processes, such as normal embryonic development, cellular signal transduction and structural integrity in microvasculature [10]. Mutations in connexins are responsible for a significant burden of heritable human syndromes, including deafness and skin disorders [6]. The human genome contains at least 20 conserved genes that encode distinct but structurally related connexin protein family [10].

Cx30 encoded by *GJB6* is an important member in connexin family which is highly expressed in epithelial, especially the palms of the hands and soles of the feet, hair follicles, nail beds and mesenchymal structures of the inner ear [2, 7, 8]. The intact size and domain of Cx30 are essential to maintain the transport of potassium ions and certain small molecules at gap junctions between cells [3, 5, 9]. In our case, the p.A88V mutation of *GJB6* introduced a highly hydrophobic residue in the transmembrane M2 domain of Cx30, which change the polarity of connexin channels and affect intercellular communication.

In year of 2000, Lamartine et al. first identified mutations (p.G11R and p.A88V) in *GJB6* could cause HED and these two variants can form intercellular channels and make functional hemichannels which may change paracrine signals [8]. Thus far, different mutation loci can jointly or independently change amino acids sequence in the Cx30 protein which lead to structural modification and abnormalities in the growth, division, and maturation of cells in the hair follicles, nails, and skin. In addition, mice model with same mutation in *GJB6* can present with similar hearing dysfunction and hyperproliferative sebaceous glands [11], which also proved the protentional pathogenic role of single nucleotide transition in *GJB6*.

Different populations may have distinct most frequent variant in *GJB6.* Notably, p.G11R and p.A88V mutations in *GJB6* were recurrently reported in Chinese Han population [3, 5]. Whether HED-related mutations in *GJB6* have its own spectrum in Chinese Han population needs further observation and investigation [2, 5]. To the best of our knowledge, p. A88V mutation was only reported in three Chinese families and we reported another family here [5, 12, 13]. In previous reports carrying p. A88V mutation, nail abnormalities in HED usually presented with short, thickened and brittle nail plates mimic pachyonychia congenita [2, 5, 12, 13]. However, fingernails of our patient have pterygium formation which may be an unusual manifestation for patients with the same p. A88V mutation described by Sukakul *et al.* [14].

Because of the rarity of HED, it is difficult to be diagnosed. Gene testing should be the most effective and accurate method in diagnosis of this congenital syndrome especially in prenatal diagnosis. Identification of mutations in *GJB6* will help those young individuals from the same family avoiding new patients born. Furthermore, mutations in other connexins, such as Cx26 (encoded by *GJB2*), can cause some overlapping features of HED including nail dystrophy, hair loss, and palmoplantar keratoderma [5, 15]. Therefore, whole-exome sequencing is needed in some individuals with HED-like characteristic cutaneous findings, especially when no mutations in *GJB6*.

Currently, there are no effective treatments for HED, and gene therapy is just a concept. However, in recent studies, fetus carrying the mutation can be identified by prenatal diagnosis, which showed prenatal diagnosis supplies a method to prevent the mutated genes from transmitting to next generation and may provide genetic base for gene treatment [2]. More studies are needed to explore the effective treatments of HED in the future.

In conclusion, this case stresses the pathogenic role of p.A88V mutation in *GJB6* and highlights the need for genetic testing in patients with characteristic cutaneous findings.

## Data Availability

availably.

## Abbreviations

HED: Hidrotic ectodermal dysplasia
GJB6: Gap junction beta 6
GJB2: Gap junction beta 2
Cx30: Connexin 30
Cx26: Connexin 26
